# The Neurometabolic Basis of Mood Instability: The Parvalbumin Interneuron Link—A Systematic Review and Meta-Analysis

**DOI:** 10.3389/fphar.2021.689473

**Published:** 2021-09-20

**Authors:** Antonello Pinna, Alessandro Colasanti

**Affiliations:** ^1^ School of Life Sciences, University of Sussex, Brighton, United Kingdom; ^2^ Department of Neuroscience, Brighton and Sussex Medical School, University of Sussex, Brighton, United Kingdom

**Keywords:** parvalbumin, Hippocampus, neuronal excitability, GABAergic interneuron, mitochondria, bipolar affective disorder, mood instability, gamma waves

## Abstract

The neurobiological bases of mood instability are poorly understood. Neuronal network alterations and neurometabolic abnormalities have been implicated in the pathophysiology of mood and anxiety conditions associated with mood instability and hence are candidate mechanisms underlying its neurobiology. Fast-spiking parvalbumin GABAergic interneurons modulate the activity of principal excitatory neurons through their inhibitory action determining precise neuronal excitation balance. These interneurons are directly involved in generating neuronal networks activities responsible for sustaining higher cerebral functions and are especially vulnerable to metabolic stress associated with deficiency of energy substrates or mitochondrial dysfunction. Parvalbumin interneurons are therefore candidate key players involved in mechanisms underlying the pathogenesis of brain disorders associated with both neuronal networks’ dysfunction and brain metabolism dysregulation. To provide empirical support to this hypothesis, we hereby report meta-analytical evidence of parvalbumin interneurons loss or dysfunction in the brain of patients with Bipolar Affective Disorder (BPAD), a condition primarily characterized by mood instability for which the pathophysiological role of mitochondrial dysfunction has recently emerged as critically important. We then present a comprehensive review of evidence from the literature illustrating the bidirectional relationship between deficiency in mitochondrial-dependent energy production and parvalbumin interneuron abnormalities. We propose a mechanistic explanation of how alterations in neuronal excitability, resulting from parvalbumin interneurons loss or dysfunction, might manifest clinically as mood instability, a poorly understood clinical phenotype typical of the most severe forms of affective disorders. The evidence we report provides insights on the broader therapeutic potential of pharmacologically targeting parvalbumin interneurons in psychiatric and neurological conditions characterized by both neurometabolic and neuroexcitability abnormalities.

## Introduction

### Mitochondrial Dysfunction and Alterations in Neuronal Network Oscillatory Activity Contribute to Affective Pathophysiology

In this review paper, we summarize and discuss the roles and reciprocal interactions of alterations in mitochondrial energy production and neuronal networks, and we propose that these mechanisms are involved in the neurobiology of mood instability. Mood instability is a subjective state characterized by oscillations of intense affect and represents a psychopathological phenotype that cuts across traditional categorical diagnostic boundaries. As such, it fits research based on classification frameworks, such as the Research Domain Classification (RDoC) (Insel et al., 2010), which investigate neurobiological mechanisms underlying clinical phenotypes that do not fully adhere to DSM diagnoses. Mood instability tends to occur in the context of several psychiatric disorders, and while it represents a pathognomonic feature of Bipolar Affective Disorder (BPAD), it has also been found in a genome-wide association study (GWAS) to have strong genetic correlations with Major Depressive Disorder (MDD) and Anxiety Disorders (Ward et al., 2020).

The neurobiological mechanisms underlying mood instability are still largely unknown; however, converging data implicate a putative role of abnormalities in mitochondria-dependent brain energy metabolism. Several genes involved in mitochondrial energy production, such as NDUFAF3, NDUFS3, PTPMT1, KBTBD4, and MTCH2, have been found associated with “mood instability” phenotype in the only available GWAS report that specifically investigated loci associated with mood instability (Ward et al., 2020). Since the majority of studies investigating bioenergetic alterations have tended to focus on categorical, traditional diagnoses, rather than broader transdiagnostic phenotypes, our review will focus primarily on reports of alterations associated with specific affective disorders associated with mood instability.

Mitochondria, which are involved in multiple functions in cellular metabolism, including macromolecule biosynthesis, nutrient catabolism, redox homeostasis, and waste management, together with brain energy metabolism alterations have been consistently found associated with a wide range of affective disorders (Marazziti et al., 2011; Holper et al., 2019; Iwata, 2019).

BPAD represents the prototypical psychiatric disorder characterized by mood instability and cyclic mood changes and is the condition, among affective disorders, with the largest evidence of underlying mitochondrial bioenergetic alterations. Evidence of mitochondria dysfunction in BPAD has progressively accumulated over the past two decades. Postmortem examinations revealed abnormal mitochondrial morphology (Cataldo et al., 2010) and markedly reduced mitochondrial complex I (MCI) levels and activity (Andreazza et al., 2010; Andreazza et al., 2013) in the prefrontal cortex (PFC) of patients with BPAD. The expression of the MCI subunit gene, NDUFV2 at 18p11, was found to be reduced in the hippocampus (Konradi et al., 2004) and in lymphoblastoid cells (Washizuka et al., 2009) of BPAD patients. Furthermore, magnetic resonance spectroscopy (MRS) studies in BPAD patients revealed lower levels of the mitochondrial-deriving amino acid N-acetyl aspartate (NAA) in the hippocampus and PFC (Yildiz-Yesilogu and Ankerst, 2006; Frey et al., 2007), increased brain lactate (Machado-Vieira et al., 2017), and alterations in phosphocreatine (Kato et al., 1994), creatine kinase reaction rate constant (Du et al., 2018), and ATP levels after stimulation (Yuksel et al., 2015). Further observations include evidence of systemic impairment in mitochondria-dependent energy production in BPAD patients [reviewed in Nierenberg et al. (2013)], including reduced intracellular pH and higher plasma lactate levels (Kato and Kato, 2000; Machado-Vieira et al., 2017; Jeong et al., 2020), higher levels of cerebrospinal fluid oxidative stress markers (Knorr et al., 2019), increased lipid peroxidation and DNA/RNA oxidative damage, and higher levels of nitric oxide (Brown et al., 2014).

Levels of oxidative stress correlate with poorer quality of life in BPAD (Nunes et al., 2018), reflecting a possible detrimental effect exerted by reactive oxygen species (ROS) on higher CNS brain functions, although this might also indicate that oxidative stress abnormalities are the consequence of poorer quality of life resulting from the disease. In fact, metabolic stress and dysfunctional brain bioenergetic processes might also be effects of psychotropic medications or of the poorer socioeconomic status deriving from a chronic and disabling psychiatric condition such as BPAD. However, we have recently reported that patients affected by primary mitochondrial disease resulting from inherited mutations of mitochondrial DNA present much higher comorbidity rates with BPAD, MDD, and general anxiety disorder (GAD) relative to the general population, with BPAD showing the strongest association which was also the most independent from the burden of neurological disability (Colasanti et al., 2020). These data in primary mitochondrial disease patients might suggest that mitochondrial and metabolic alterations might be a cause for the development of severe affective syndromes rather than an effect.

The role of bioenergetic deficits in BPAD is also strengthened by preliminary clinical evidence suggesting beneficial effects of treatments with mitochondrial modulators such as coenzyme Q10 (CoQ10) or Methylene Blue in improving depressive or cognitive symptoms in BPAD patients (Alda et al., 2017; Mehrpooya et al., 2018).

The role of mitochondrial abnormalities is evident also in the pathophysiology of MDD, as illustrated by both rodent and human studies (Martins-de-Souza et al., 2012; Weger et al., 2020). Preclinical studies demonstrated that the exposure to chronic mild stress causes mitochondrial ultrastructure damage and inhibition of the oxidative phosphorylation complexes, resulting in reduced mitochondrial respiration rates in mice PFC (Gong et al., 2011). The lack of NAD(P)+ transhydrogenase, an essential enzyme for energy-linked reactions in the mitochondrial matrix, was found to induce depressive-like behavior in mice (Francisco et al., 2020). *Ex vivo* human studies showed reduced ATP and altered levels of proteins associated with energy metabolism in the dorsolateral prefrontal cortex in MDD patients (Martins-de-Souza et al., 2012). Genome-wide transcriptomic analyses reported that alterations in oxidative phosphorylation genes in chronic restraint stress mice models were similar to those found postmortem in MDD patients’ brain (Weger et al., 2020).

Mitochondrial dysfunctions are suggested also by several models of anxiety (Filiou and Sandi, 2019) showing imbalance in oxidative stress in both clinical (Arranz et al., 2007; Ozdemir et al., 2009) and preclinical models (Brocardo et al., 2012; Hollis et al., 2015) [summarized by Krolow et al., (2014)]. In mouse models, decreasing mitochondrial levels of Bcl-2, a mitochondrial function modulator, induces anxious behavior (Einat et al., 2005).

Alongside mitochondrial dysfunction, the altered oscillatory activity of neuronal networks, indicative of alterations in neural synchrony, is another neurobiological abnormality shared by all major neuropsychiatric disorders with affective manifestations. Although changes in oscillations in multiple frequency bands have been reported, we hereby focus on fast-oscillations in the frequency range of 30–120 Hz, i.e., gamma oscillations. Gamma oscillations are involved in several high-order brain functions, such as attention (Jensen et al., 2007), pain (Schultz et al., 2012), object recognition (Keil et al., 1999), learning (Sederberg et al., 2007), and long-term memories formation (Axmacher et al., 2006; van Vugt et al., 2010), and are particularly affected in mood disorders (Stujenske et al., 2014; Canali et al., 2017; Murphy et al., 2019). In BPAD, gamma oscillations from frontal and temporal regions are reduced compared to controls, as shown by EEG (Canali et al., 2017) and magnetoencephalography (MEG) (Lee et al., 2011; Liu et al., 2012) studies, respectively. Interestingly, gamma oscillation alterations were found to persist despite successful treatment of BPAD depressive episodes, indicating that changes in gamma synchronization do not directly contribute to symptoms manifestation and that the observed reduction of gamma oscillation might represent a trait, rather than state, marker of BPAD (Canali et al., 2017).

Findings of oscillatory alterations have also been reported for MDD, including abnormal modulation of gamma oscillatory activity during working memory encoding and maintenance in frontal regions using EEG (Murphy et al., 2019) and reduced gamma oscillations after transcranial magnetic stimulation (TMS) in frontal regions compared to controls (Canali et al., 2017), which was also confirmed in animal models of depression (Sauer et al., 2015).

Some preclinical evidence suggested alterations of gamma oscillations in anxiety, showing decreased gamma oscillations in the basolateral ganglia and medial PFC (mPFC) in mice during fear expression (Stujenske et al., 2014), while other studies reported increased gamma oscillations in experimental models of anxiety (Miskovic et al., 2010; Schneider et al., 2018).

In summary, the evidence summarized above indicates that both mitochondrial and network oscillatory alterations, among other pathological features, are implicated in the pathogenesis and pathophysiology of major psychiatric disorders associated with mood dysregulation and might be candidate mechanisms underlying mood instability, which is one of the most prominent features of severe forms of BPAD.

### Parvalbumin GABAergic Interneurons Are Involved in Neuronal Networks Dysfunction and Are Vulnerable to Brain Metabolism Dysregulation

Cortical network operations depend on complex interactions of highly interconnected and dynamic microcircuits composed of glutamatergic excitatory projection neurons and a multitude of local GABAergic interneurons that “sculpt” these networks and regulate the flow of neuronal signals (Tremblay et al., 2016). GABAergic inhibition is a multifaced function that coordinates the action of principal cells by countering excitation, exerting selective filtering of synaptic excitation, and modulating the gain, timing, tuning, bursting of excitatory cells firing. Inhibition by GABA interneurons allows stability and transient autonomy of principal cells populations, through different forms of highly specialized inhibitory microcircuits, provided by different interneuron subtypes. The main types of GABAergic inhibitory microcircuits are feedforward circuits (where interneurons receive excitatory inputs from external sources and in turn inhibit the principal excitatory neuron); feedback circuits (where interneurons receive excitation from principal cells and, in turn, inhibit them); and lateral inhibitory circuits (where an assembly of principal cells suppress the activity of another assembly of principal cells through the excitation of inhibitory interneurons).

A subtype of GABAergic interneurons, namely, fast-spiking parvalbumin interneurons (PV-INs), constitutes the largest IN population in the neocortex and is of particular relevance to the pathophysiology of brain disorders associated with mitochondrial and bioenergetic dysfunction due to their particularly high metabolic demands.

PV-INs are characterized by the expression of the Ca^2+^-binding protein parvalbumin, whose presence is restricted to GABAergic interneurons (Celio, 1986; Kosaka et al., 1987). PV-INs can be subdivided into chandelier (or axoaxonic) cells and fast-spiking basket cells. The latter display a fast-spiking phenotype, consisting in their ability to generate high-frequency spikes of action potentials (APs) (>50 Hz at 22°C and >150 Hz at 34°C) during continuous current injection *in vitro*, without accommodation (Cauli et al., 1997; Rudy and McBain, 2001; Hu et al., 2014; Hu et al., 2018).

PV-INs’ somata possess the largest share of inhibitory terminals of all GABAergic boutons (Gulyás et al., 1999) and innervate numerous excitatory target cells close to the sites of APs generation. Their morphological structure with multiple dendrites allows them to receive inputs from several afferent pathways, mainly convergent excitatory inputs from principal neurons (more than 90% of afferents on PV-INs are excitatory) (Gulyás et al., 1999) and a small proportion of inhibitory inputs, mainly from other GABAergic interneurons, such as somatostatin interneurons (SST-INs) (Pfeffer et al., 2013) or other PV-INs. By mostly receiving strong excitatory inputs, and by innervating pyramidal neurons at the soma, PV fast-spiking basket cells are strategically positioned to exert both feedforward and feedback inhibition and gain control (Espinoza et al., 2018; Scudder et al., 2018) to excitatory outputs. Their fast inhibitory control is fundamental in restricting and refining AP firings of postsynaptic neurons, enabling temporal processing and synchronized firing of excitatory neuronal populations (Isaacson and Scanziani, 2011; Kee et al., 2015) which results in an optimal excitation and inhibition balance in cortical circuits (Ferguson and Gao, 2018). PV-INs inhibitory action on excitatory neurons is implicated in the generation and maintenance of synchronized oscillatory network activity, in particular gamma oscillations (Csicsvari et al., 2003; Sohal et al., 2009; Veit et al., 2017) in most brain areas. These depend mainly on fast synaptic inhibition by PV-INs targeting the perisomatic domain of excitatory pyramidal cells (Kim et al., 2016; Chen et al., 2017; Antonoudiou et al., 2020). However, it is worth considering that, in specific cortical areas (e.g., V1), other GABAergic interneuron subtypes, in particular SST-INs, play an important role in the generation of lower frequency band oscillations (up to 30 Hz), including those in the lowest end of the “typical” gamma spectrum (∼30 Hz) (Veit et al., 2017; Antonoudiou et al., 2020).

Although PV-INs are widely distributed throughout the whole mammalian brain, their density varies throughout the brain. Throughout the neocortex, these neurons are mostly present in middle layers, especially layers II, III, and IV, consistent with their role in organizing the activity of microcircuits, while they are completely absent in layer I (Zhu et al., 2018). In the cortex, they innervate both pyramidal cells and other interneurons [reviewed in Tremblay et al. (2016)]. There is a high density of PV-INs also in the hippocampus. In the CA1 region, they mainly innervate the soma and dendrites of pyramidal cells (99% of output synapses) with a minority of outputs contacting with other interneurons, while in the dentate gyrus, they mainly innervate granule cells [reviewed by Pelkey et al. (2017)].

For their functions, PV-INs require an abundant and continuous supply of oxygen and glucose through optimal mitochondrial functions, and their activity is especially susceptible to brain energy level changes (Kann, 2016). PV-INs are continuously active in every cycle of gamma oscillations (Hájos et al., 2004; Gulyás et al., 2010; Tukker et al., 2013). The extremely high energy demand of fast-spiking signaling requires mitochondria to generate high adenosine triphosphate (ATP) levels. This is consistent with the notion that AP transmission and postsynaptic receptors’ ion flux are the most ATP-consuming neuronal activities (Attwell and Laughlin, 2001) and PV-INs generate fast and brief APs [which are considered per se even more energy expensive than normal, as suggested by Carter and Bean (2009)] at elevated frequencies for extended periods of time (Gulyás et al., 1999; Jonas et al., 2004). Furthermore, PV-INs increased metabolic demands (Jiang et al., 2013) might be not only due to PV-INs fast-spiking activity but also to their dense excitatory innervation (Gulyás et al., 1999). Such heavy reliability on mitochondrial energy metabolism is also represented by a greater number of mitochondria compared to other neurons (Gulyas et al., 2006). *In vitro* evidence showed that gamma oscillations use mitochondrial oxidative capacity near limit (Kann et al., 2011; Huchzermeyer et al., 2013) and that even subtle mitochondrial impairments might result in disruption of gamma oscillations as demonstrated by the rapid decline of hippocampal gamma oscillations’ power observed in hypoxic conditions (Huchzermeyer et al., 2008) or after pharmacologically induced mitochondrial impairments, inhibiting MCI’s functions (Whittaker et al., 2011).

## Alterations of Parvalbumin GABAergic Interneurons in Bipolar Disorder: A Meta-Analysis

PV-INs deficits have been implicated in the neurobiology of a broad range of neuropsychiatric disorders, including schizophrenia, where the putative mechanistic contribution of cortical GABA neurons and their dysfunctions, including downstream effects, have been well characterized (Lisman et al., 2008; Lewis et al., 2012; Kaar et al., 2019). Abundant PV expression and consequent efficient Ca^2+^ buffering by PV are prerequisites for an adequate inhibition of cortical networks. The absence of PV in GABAergic interneurons modifies the dynamics of the inhibitory control at the local level, with important implications on the overall resulting excitation/inhibition balance in cortical microcircuits (Schwaller et al., 2004; Caballero et al., 2020). However, the relationship between PV expression and activity of PV-INs neurons appears complex, as opposite results have been observed in full PV knockout mice, where the complete absence of PV caused increased inhibitory postsynaptic currents through increased facilitation of GABA release (Vreugdenhil et al., 2003). These discrepant findings might reflect the hyperexcitability of INs in PV knockout mice, possibly a compensatory process resulting from the complete absence of PV since the embryonic stages (Caballero et al., 2020).

In the following sections of this review, we will present arguments in support of a possible role of PV-INs in mechanisms underlying mood instability characteristic of chronic and severe mood disorders such as BPAD, which, as summarized in the previous *Mitochondrial Dysfunction and Alterations in Neuronal Network Oscillatory Activity Contribute to Affective Pathophysiology*, are associated with both brain metabolism dysregulation and neuronal networks’ dysfunction. To provide empirical support to this hypothesis, in the next paragraph, we report the results of a meta-analysis of PV-INs loss or dysfunction in the brain of patients with BPAD.

Evidence on PV-INs alterations in BPAD is not unequivocal with individual studies showing either marked, mild, or no alterations of PV-INs density or number in BPAD patients. We therefore performed a meta-analysis of studies assessing PV-INs alterations in the brain of patients with BPAD and separately reported data on PV-INs total number, PV-INs cells densities, and PV mRNA levels.

## Methods

### Search Strategy

A standardized search was conducted on Medline/PubMed electronic database. The following keywords were used: parvalbumin AND bipolar disorder [“parvalbumins”(MeSH Terms) OR “parvalbumins”(All Fields) OR “parvalbumin”(All Fields)] AND [“bipolar disorder”(MeSH Terms) OR [“bipolar”(All Fields) AND “disorder”(All Fields)] OR “bipolar disorder”(All Fields)] until January 2021. We used only those stated keywords because we identified them as being the most inclusive for our aim. We explored the use of alternative search strategies including related keywords, such as “calcium-binding proteins” AND “bipolar disorders” or parvalbumin AND bipolar affective disorder but these searches did not extract any additional relevant article that had not been already identified with the use of the previously stated keywords.

#### Study Selection

**FIGURE 1:**
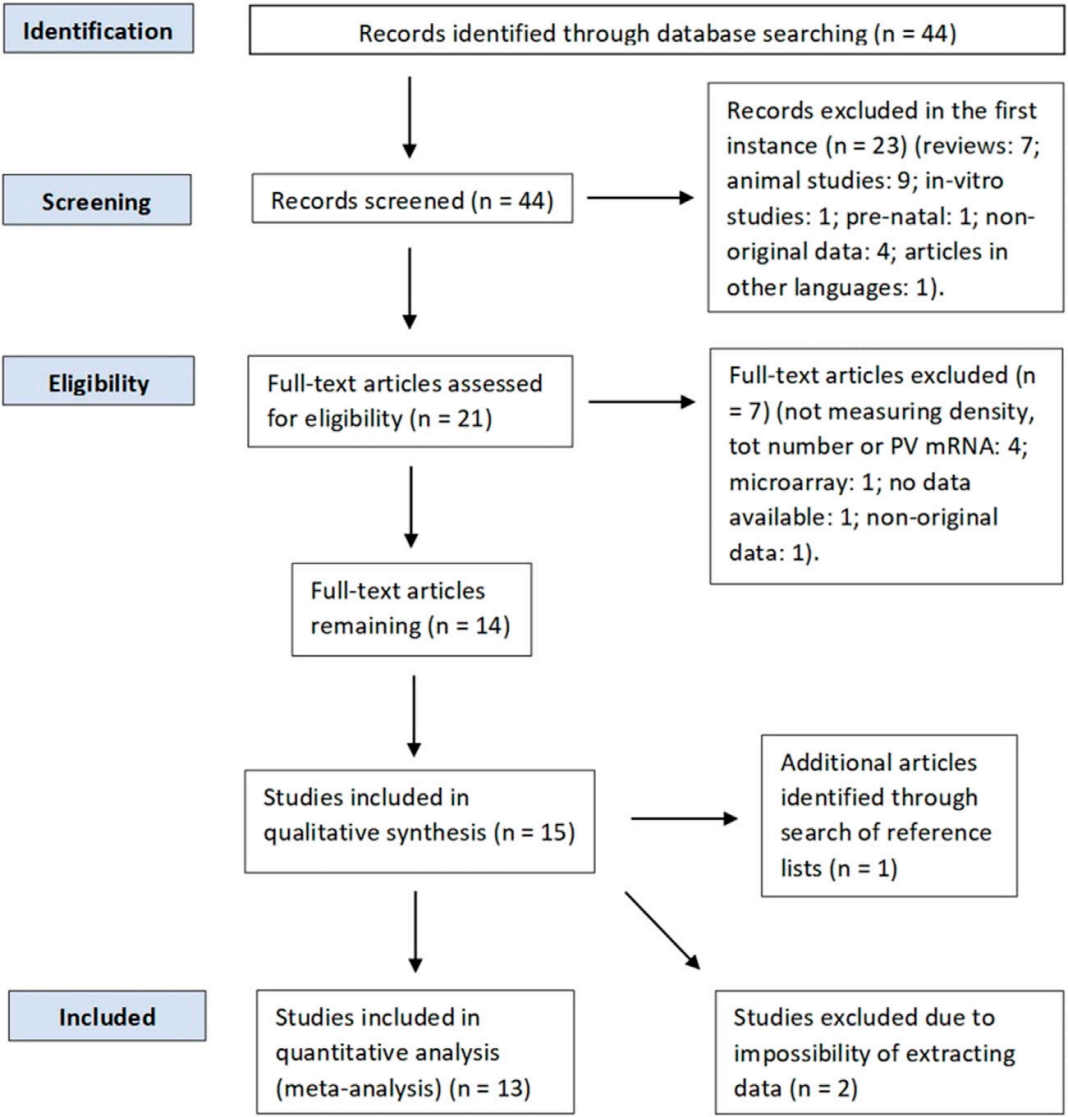
Meta-analysis PRISMA flow diagram.

Inclusion criteria were original studies reporting postmortem findings of either PV-INs density, total number, or PV mRNA levels in any brain region available; studies analyzing BPAD patients (BPAD type I or II) and a control group. Exclusion criteria were prenatal studies, animal studies, *in vitro* studies, articles in a language different than English, review articles, studies without a control group, articles that were not published in a peer-reviewed journal, and articles without available data (either numerical or graphical). Studies that measured GABA-related transcripts or markers but not PV mRNA were not included. In addition, we also excluded microarray studies (n = 1) (Gandal et al., 2012). Although they have been used for comparison with the data we analyzed, studies that reanalyzed existing datasets of multiple transcriptomic studies, investigating PV marker gene profile (MGP) (Toker et al., 2018), or studies that reanalyzed data from published and unpublished studies (Torrey et al., 2005) were not included in the meta-analysis and were classified as studies reporting nonoriginal data. However, four studies (Alcaide et al., 2019; Beasley et al., 2002; Reynolds et al., 2002; Zhang and Reynolds) analyzed brain samples obtained from the same brain bank (the Stanley Foundation Neuropathology Consortium brain collection) [details of such brain bank are explained by Torey et al. (2000)] and we still decided to include those studies in the quantitative analysis, as they all reported different results, and most of them assessed PV-INs in different brain areas from one another. Study selection was performed according to PRISMA guidelines (Liberati et al., 2009), and a PRISMA diagram of the full literature search is displayed in Figure 1.

### Data Extraction

In our study, we included three outcome measures: 1) the effect size for the difference in PV-INs cell density [either in cells/mm^2^ (or cells. mm^−2^) and cells/mm^3^], 2) the effect size for the difference in PV-INs total number between BPAD patients and controls, and 3) the effect size for the difference in PV mRNA expression ratio (reported as 2^−dCT^) per region between BPAD patients and controls. For each study, we extracted the mean and standard deviation of the outcome measures listed, for all brain regions analyzed, in BPAD patients and healthy controls (HCs), using the available published data. When data were not presented in the text but displayed only with graphs and plots, we either calculated and extracted values manually from the graph or used WebPlotDigitizer (https://automeris.io/WebPlotDigitizer) depending on the resolution of the image available. If cell density, total number, or mRNA was measured across different layers for neocortical areas (e.g., Beasley et al., 2002), data were combined into one mean value for the region, and the standard deviation was calculated among the mean values of the layers, for each group (BPAD or HCs). The same procedures have been followed for studies that analyzed PV-INs in different subregions of the same brain area (e.g., Pantazopoulos et al., 2010). This method should not influence the statistical significance or the overall trend of our measurements, because in all studies analyzed (and in which was performed this procedure of combining data from different layers or subregions of the same brain area), all values in each layer/or subregion were lower in the BPAD group compared to HCs, apart from Sakai et al. (2008), in which in some PFC (BA9) layers, PV-INs density values were slightly higher in the BPAD group. When values reported were from different layers in the cortex [e.g., Beasley et al. (2002); Reynolds et al. (2002)], layer I was excluded from the combined analysis because of the absence of PV-INs in layer I of the cortex (Zhu et al., 2018). In one case (Wang et al., 2011), in addition to the size of the sample trial, authors reported only the median and range values; in this case, mean and SD values have been estimated using the formulas described by Hozo et al. (2005), respectively, $\bar{x}\;\approx \;\frac{a+2m+b}{4}$ and $S^{2}=\frac{1}{12}\left(\frac{{\left(a-2m+b\right)}^{2}}{4}+{\left(b-a\right)}^{2}\right)$, in which *m* is the median value and *a* and *b* are the low and high ends of the range, respectively.

### Data Analysis

**FIGURE 2:**
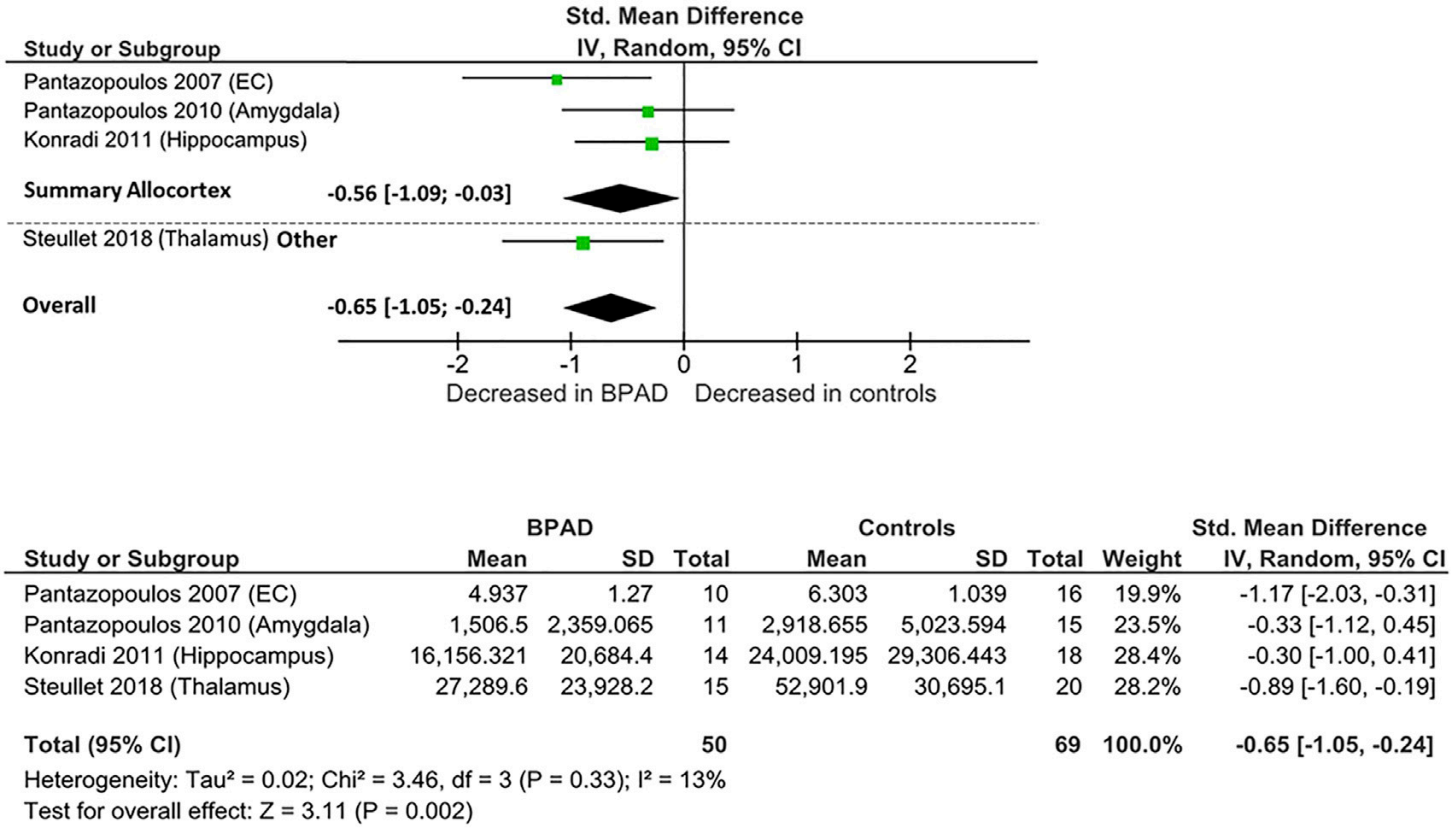
Forest plot on PV-INs total number: allocortical areas, others, and overall comparison.

**FIGURE 3:**
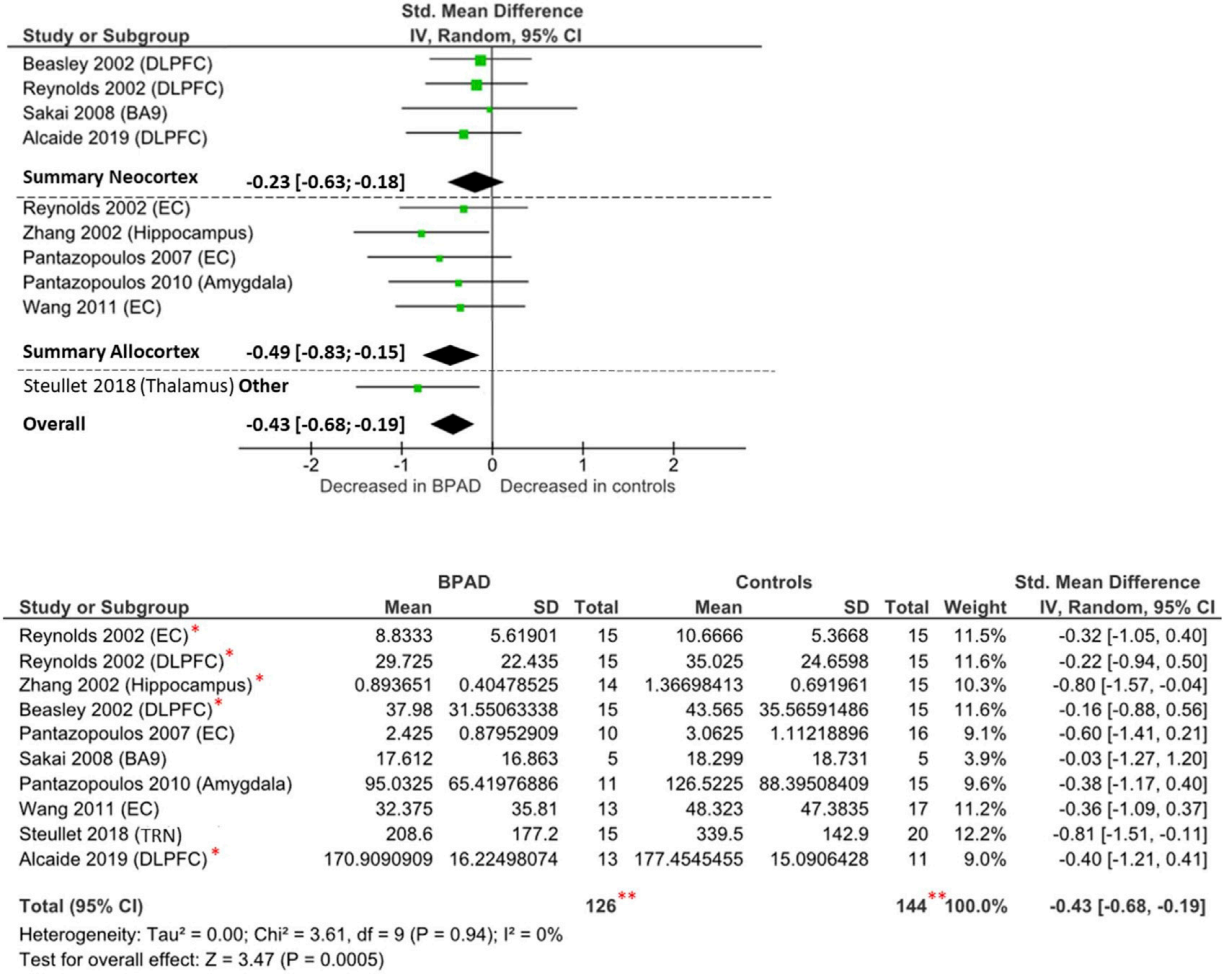
Forest plot on PV-INs cell density: neocortical, allocortical, other, and overall comparison. **The total number of participants displayed is twice the number of subjects studied by Reynolds et al. (2002), as they performed their analysis in both dorsolateral prefrontal cortex and entorhinal cortex and the repetition of the same cohort of brain samples, which has been analyzed by several research groups (Zhang 2002; Beasley 2002; Alcaide 2019). *These studies analyzed the same brain samples obtained from the Stanley Foundation Neuropathology Consortium brain collection (Torrey et al., 2000).

**FIGURE 4:**
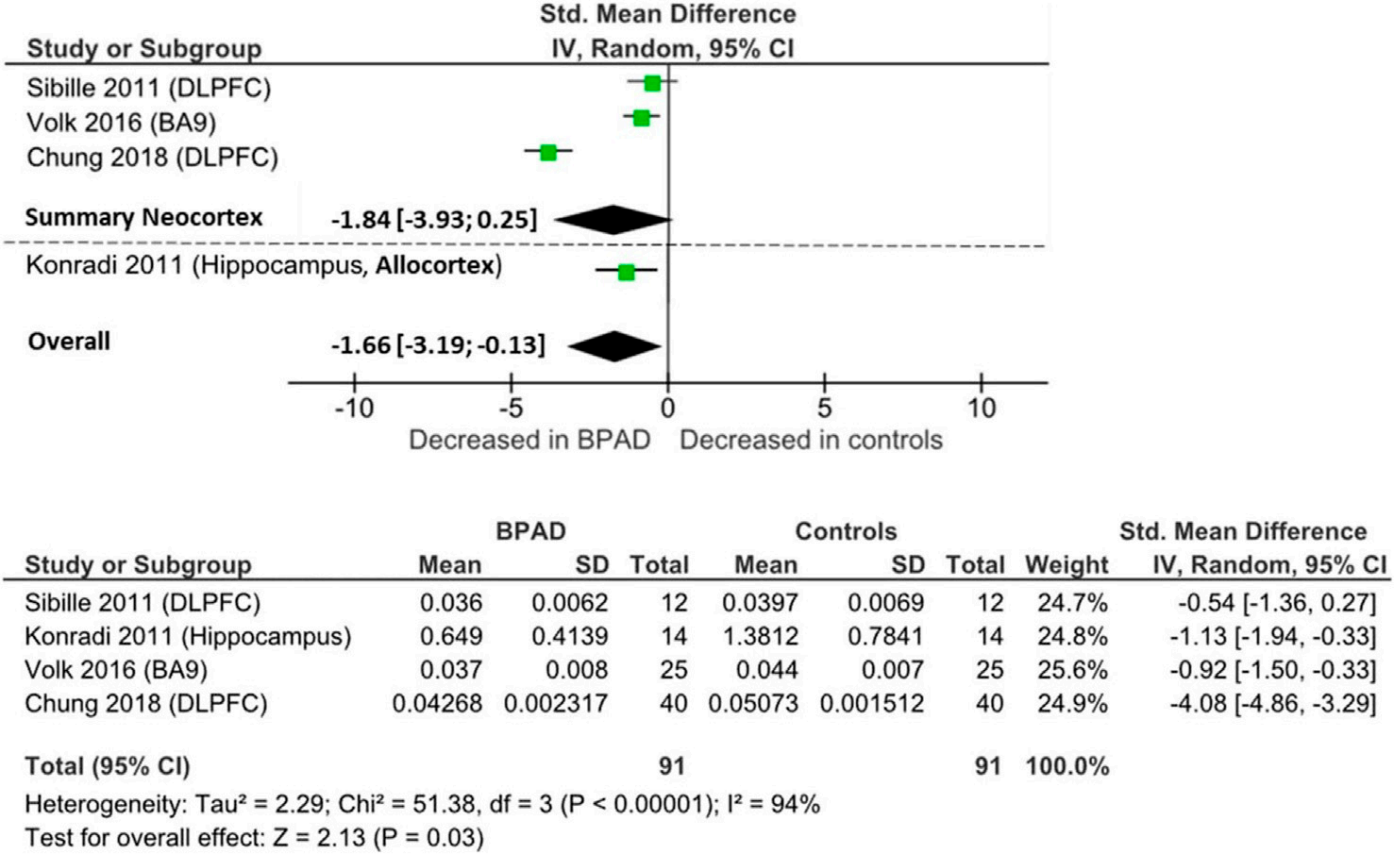
Forest plot on PV mRNA expression: neocortical, allocortical, and overall comparison.

RevMan software (Review Manager 5.4.1) was used to perform the meta-analysis and to create the forest plot seen in Figures 2, 3, 4. Group size, mean, and standard deviation were used to determine the standardized mean difference (Cohen’s D values). An inverse variance, random-effects meta-analytic model was used, with 95% confidence intervals; we therefore did not assume homogeneity of effects. Heterogeneity was measured by calculating I^2^, Tau^2^, Chi^2^, and a *p* value < 0.05. Then, a Z test, with a respective *p* value, was performed to test the significance level of the overall effect. In addition to analyzing together all brain areas divided only for the outcome measure, we performed subgroups analysis whenever possible, grouping and analyzing together studies according to the main part of the telencephalon they assessed, specifically neocortex or allocortex, keeping the subdivision dependent on the outcome measure. However, one study measured PV-INs cell density and total number in a noncortical brain area, the thalamic reticular nucleus (TRN), which is part of the diencephalon and is characterized by a different cell layer organization. Therefore, it could not be included in neither of the two types of cerebral cortex. We subgrouped studies according to the type of cortex analyzed, considering the differential expression of PV-INs across different brain areas and the potential different vulnerability of different brain areas to neurometabolic stress. Given the relative scarcity of studies that assessed PV-INs in postmortem patients, further subgrouping of studies at individual brain region level has not been possible.

Due to the diversity of brain areas and of unit of measurement (such as cells/mm^3^ and cells/mm^2^), we calculated the standardized mean difference, instead of the normal mean difference, in order to convert study results to a standardized effect size and to compare them together. The assessment of publication bias, using the Egger’s test for funnel plot asymmetry (Egger et al., 1997), would have been feasible only for the subgroup analysis (neo- or allocortex), as otherwise, in the overall comparisons, such test would have possibly led to asymmetry, not inducible to real publication bias but instead mostly related to the selectivity of outcomes measures selected for analysis (Egger et al., 1997; Sterne et al., 2011), and by the inclusion of different brain areas. Subgroup tests for asymmetry could not be conducted, because the sample size of studies included in each subgroup was not bigger or equal to 10 (Sterne et al., 2011).

## Results

### PV-INs Total Number

Four postmortem studies compared the total number of PV-INs between BPAD patients and control subjects, resulting in a total sample of 50 BPAD patients’ and 69 controls’ brains (Figure 2). Of these, three studies examined PV-INs’ number in allocortical structures (hippocampus, entorhinal cortex (EC), and amygdala) and one analyzed TRN. As displayed in Supplementary Table S1, in the allocortex, PV-INs total number appear to be significantly reduced in the hippocampus (CA1,2/3,4) (*p* = 0.029) (Konradi et al., 2011) and in the whole EC (*p* = 0.02) (Pantazopoulos et al., 2007), while no significant differences have been noticed in amygdala nuclei, specifically lateral nucleus (*p* = 0.51), basal nucleus (*p* = 0.94), accessory basal (*p* = 0.99), and cortical nuclei (*p* = 0.28) (Pantazopoulos et al., 2010). Significant PV-INs total number reductions have been observed also in the TRN (*p* < 0.0001) (Steullet et al., 2018).

When these studies were pooled together, there was an overall significant reduction in PV-INs total number in BPAD patients compared to controls (Cohens’ d = −0.65; z = 3.31; *p* = 0.002; 95% confidence interval (CI): −1.05 to −0.24) (Figure 2). The I^2^, Tau^2^, and Chi^2^ tests revealed small heterogeneity (I^2^ = 13%; Tau^2^ = 0.02; Chi^2^ = 3.46). After dividing studies according to the type of cortex analyzed (Figure 2), we observed a reduction of PV-INs total number in allocortical areas in BPAD brains compared to controls (Cohens’ d = −0.56; z = 2.06; *p* = 0.04; CI: −1.09 to −0.03) with a small heterogeneity (I^2^ = 28%; Tau^2^ = 0.06; Chi^2^ = 2.79).

#### PV-INs Cell Density

Ten studies have examined the PV-INs density in the brain of BPAD patients and compared these to control subjects, resulting in a total number of 111 BPAD patients’ brains and 125 controls’ brains (Figure 3). Of these, five studies examined allocortical structures (EC, hippocampus, and amygdala), while four studies analyzed neocortical structures (DLPFC and BA9) and one study assessed the TRN.

Findings were heterogenous also within the same brain structures (Supplementary Table S2): reduced PV-INs density has reported in the EC (*p* < 0.05) in both Pantazopoulos et al. (2007) and Wang et al. (2011), but not in Reynolds et al. (2002) (*p* > 0.05). In the allocortex, reductions have been noticed in the hippocampus, but only from the CA1 region (*p* < 0.05) (Zhang et al., 2002), and similar differences have been observed in the amygdala nuclei, but only from the lateral nucleus (*p* = 0.03) (Pantazopoulos et al., 2010). Other relevant differences have been reported in TRN (*p* < 0.0001) (Steullet et al., 2018). There were no significant changes reported in any of the two neocortical areas examined (DLPFC and BA9) (Beasley et al., 2002; Reynolds et al., 2002; Sakai et al., 2008; Alcaide et al., 2019). Another study (not included in the meta-analysis due to unavailable raw data) reported no significant difference in PV-INs density in the anterior cingulate cortex (ACC) (Cotter et al., 2002).

Overall, comparing all brain areas together, PV-INs cell density appears to be reduced in BPAD brains compared to controls (Cohens’ d = −0.43; z = 3.47; *p* = 0.0005; 95% CI: −0.68 to −0.19) (Figure 3). The I^2^, Tau^2^, and Chi^2^ tests revealed small heterogeneity (I^2^ = 0%; Tau^2^ = 0; Chi^2^ = 3.61). After dividing studies according to the type of cortex analyzed (Figure 3), we observed a reduction of PV-INs cell density in allocortical areas (Cohens’ d = −0.49; z = 2.81; *p* = 0.005; 95% confidence interval CI: −0.83 to −0.15) but not in neocortical areas (Cohens’ d = −0.23; z = 1.09; *p* = 0.27; 95% CI: −0.63 to 0.18). In both allocortex and neocortex subclassifications, the I^2^, Tau^2^, and Chi^2^ tests revealed small heterogeneity (I^2^ = 0%; Tau^2^ = 0; Chi^2^ = 1.11 and I^2^ = 0%; Tau^2^ = 0; Chi^2^ = 0.30, respectively).

#### PV mRNA

Four postmortem studies investigated PV mRNA levels, resulting in a total sample of 91 BPAD patients’ brains and 91 controls’ brains for comparison in the quantitative analysis. PV mRNA levels were lower in neocortical areas in BPAD, specifically PFC area 9 (*p* = 0.001) (Volk et al., 2016) and DLPFC (*p* < 0.05) (Chung et al., 2018; Sibille et al., 2011) (Supplementary Table S3), while Fung et al., 2014 found no changes between the two groups in either DLPFC or orbitofrontal cortex. mRNA levels were reduced in the hippocampal CA2/3 and CA4 areas (*p* < 0.05), but not in the CA1 region (NS) (Konradi et al., 2011).

When pooling all brain areas together, there was an overall significant reduction in BPAD patients compared to controls (Cohens’ d = −1.66; z = 2.13; *p* = 0.03; 95% CI: −3.19 to −0.13) (Figure 4 **)**. In this case, the I^2^, Tau^2^, and Chi^2^ tests revealed considerably high heterogeneity (I^2^ = 94%; Tau^2^ = 2.29; Chi^2^ = 51.4). After dividing studies according to type of cortex, in neocortical areas, we only observed a trend of reduction of PV mRNA in BPAD brains compared to controls (Cohens’ d = −1.83; z = 1.73; *p* = 0.08; 95% CI: −3.93 to 0.25) (Figure 4 **)**. The I^2^, Tau^2^, and Chi^2^ tests revealed considerably high heterogeneity in this case too (I^2^ = 96%; Tau^2^ = 3.26; Chi^2^ = 49.99). In allocortical areas, specifically in the hippocampus, there was an overall significant reduction in PV mRNA levels in BPAD compared to controls in hippocampal subareas CA2/3 and CA4, but not in the CA1 region.

## Discussion

The results of our meta-analysis showed significant changes in PV-INs in BPAD; however, these varied depending on the brain areas analyzed. Specifically, we found reductions in PV-INs total number and in PV-INs cell density in allocortical areas and in the TRN in BPAD compared to controls, but not in PV-INs cell density and PV mRNA in the neocortex. It is important to note that the reduced presence of PV-INs neurons might not only reflect neuronal downregulation but might be the result of either improper neurodevelopment (e.g., neuronal immaturity) or reduced PV expression, which in turn affects PV-INs function (Caballero et al., 2020).

Although there was an overall reduction when all brain areas were analyzed together, our findings suggest that the most obvious deficits of the PV-INs system in BPAD patients occurred in allocortical areas. The reason behind this specificity is potentially due to the elevated oxygen metabolism requirements of some allocortical areas, such as the hippocampus (Cooper et al., 2015; Maiti et al., 2008) suggesting a higher susceptibility for dysfunction in conditions of suboptimal metabolic substrates availability. Alternatively, methodological explanations could underlie the reduction in PV-INs measures observed in allocortical but not neocortical areas. Meta-analyzing studies that assessed cell density or cells’ total number can lead to several potential sources of heterogeneity. In general, methodological differences, such as the method of tissue fixation (Ahram et al., 2003; Hoetelmans et al., 2001) or labeling and microscopy techniques, can result in highly heterogeneous data, although they are still comparable using a standardized effect size. Even though all the included studies followed rigorous protocols for a reliable diagnosis of BPAD, some studies included both BPAD types I and II and some others did not specify the BPAD type diagnosed. In addition, a meta-analysis of postmortem studies implies potential differences in the clinical characteristics of the patients studied, including substance or alcohol misuse and pharmacological treatments. In addition, the reliability of measures could have been influenced by potential differences in the microbiological composition or by different degrees of tissue shrinkage of brain samples analyzed. The manual average of data recorded from specific brain layers in the neocortex or subregions in some allocortical areas might lead to slight differences compared to results obtained from studies that performed their analysis as a whole region but excluded some layers from their analysis (Alcaide et al., 2019). The precision of data reported might be weakened also by the manual or automatic extrapolation of data from graphs, due to the unavailability of raw data in the paper. It is also worth considering that the apparently lower PV-INs total number and cell density might result from reduced PV expression that renders some PV-INs difficult to detect using fixation methods that tend to lower the immunoreactivity of the samples (such as paraffin embedding) (Stan and Lewis, 2012). However, it is unlikely that this has biased our results: the analysis of fixation methods employed in studies assessing the total number and cell densities (reported in Supplementary Table S1, 2) indicates that findings were not dependent on the methods used for fixation. The use of low magnification imaging might also lead PV cells with lower PV expression to be undetectable, which led to apparent reductions in PV-INs density observed in conditions with low PV expression such as schizophrenia (Enwright et al., 2016). This could have also potentially affected findings in BPAD patients. The use of a detection threshold for levels of PV would have been advisable to improve the reliability of such immunocytochemistry postmortem analysis when low magnification is used.

The finding of significantly downregulated PV-INs in BPAD patients is in agreement with studies that investigated the MGP of PV-INs of 15 existing datasets of multiple transcriptomic studies, reporting an overall reduction of PV-INs expression in BPAD patients compared to controls (Toker et al., 2018). Although MGPs do not directly measure cell numbers, they are reliable representations of the abundance of specific cell types across samples (Mancarci et al., 2017). Similar PV-INs deficits have been suggested also by microarray studies, reporting significant PV expression reductions in BPAD patients compared to controls (Gandal et al., 2012). These outcomes are finally confirmed also by animal models, that showed PV downregulation in Brd1^+/−^ mice (Qvist et al., 2018), and such mutations have been linked to BPAD susceptibility (Severinsen et al., 2006).

Similar deficits of PV-INs have been observed in a variety of conditions that have not been covered here in detail. These, for example, include schizophrenia (Beasley and Reynolds, 1997; Kaar et al., 2019), which shares phenomenological similarities with BPAD, or epilepsy (Medici et al., 2016), which instead manifests with symptoms different from those of BPAD. Although it is not possible to draw a precise conclusion on why such diverse clinical outcomes may originate from common underlying neurobiological deficits, it is possible that multiple factors play a role, including a different extent of PV-INs reduction, the possible presence in other diseases of further concomitant deficits affecting other cellular types, a different regional distribution of deficits, and the temporal onset of these deficits that might differ across conditions, and hence might have a differential effect on neurodevelopmental processes.

Another important aspect to consider for the interpretation of our findings is that cell types other than PV-INs have been found to be reduced in BPAD and that BPAD is associated with a more generalized deficit of the GABAergic system beyond PV-INs, including, for example, reduced plasma and cerebrospinal fluid (CSF) GABA levels (Berrettini et al., 1983) and reduced GABAergic activity markers, such as glutamate decarboxylase 67 (GAD67) (Guidotti et al., 2000; Fatemi et al., 2005; Buttner et al., 2007). A general reduction on GABAergic interneurons was observed through the analysis of proteins such as Reelin, preferentially secreted by GABAergic interneurons in rats (Alcàntara et al., 1998) and primates (Rodiguez et al., 2000), with a specific reduction of Reelin mRNA by about 50%, and a reduction of the density of Reelin-immunopositive neurons by 25/30% in the PFC of BPAD patients compared to HCs (Guidotti et al., 2000). Also, a reduced cell density of other subpopulations of GABAergic interneurons, such as somatostatin interneurons, was observed in the EC (Wang et al., 2011) and amygdala (Pantazopoulos et al., 2017) of BPAD patients compared to HCs. Other findings included decreased somatostatin interneurons’ total number in the amygdala (Pantazopoulos et al., 2017) and hippocampus (Konradi et al., 2011) and a significant lower somatostatin mRNA in DLPFC, orbitofrontal cortex, and hippocampus of BPAD patients, relative to control subjects (Konradi et al., 2011; Sibille et al., 2011; Fung et al., 2014). Other studies investigated other types of interneurons, calbindin, and calretinin, overall suggesting a reduction of these cells in BPAD although the results appear still inconclusive (Beasley et al., 2002; Cotter et al., 2002; Reynolds et al., 2002; Sakai et al., 2008; Fung et al., 2014).

Taking into account other confounding factors that might contribute to the observed deficits, some animal studies report an age-related PV-INs decline (Steullet et al., 2018; Ueno et al., 2018; Rogalla and Hildebrandt, 2020), while others report an increase in PV-INs cell number in aged mice compared to young mice, showing the same outcomes in rats and gerbils (Ahn et al., 2017). This dependence has been analyzed also in clinical studies, without showing any age-related PV-INs changes either in normal conditions (Bu et al., 2003), or in BPAD (Pantazopoulos et al., 2010; Wang et al., 2011; Steullet et al., 2018). Although this evidence suggests a general impairment of GABAergic interneurons in BPAD, the data that report reductions of other GABAergic interneurons’ subtypes appear to be still inconclusive, and the other potential confounding factors did not appear to have any significant influence on the reduction we detected, strengthening the idea of an underlying mechanism in BPAD patients, which selectively targets PV-INs.

Mitochondrial abnormalities are among the possible mechanisms responsible for PV-INs loss and dysfunction, which ultimately disrupt the regulatory interneurons function and lead to alterations in cortical excitability and neural networks function. Converging evidence indicates that insufficient availability of energy substrates and inefficient mitochondrial oxidative phosphorylation, and resulting oxidative stress, might be plausible causes for PV-INs loss and dysfunction. Perinatal hypoxia-ischemia has been found associated with marked loss of cortical PV-INs (Fowke et al., 2018). An exponential production of ROS by dysfunctional mitochondria appears to be directly linked to PV-INs dysfunction and downregulation. The unique metabolic demands of PV-INs might suggest a particular susceptibility to oxidative stress (Steullet et al., 2017) and antioxidants depletion-induced oxidative stress, leading to PV immunoreactivity downregulation in PV-INs (Cabungcal et al., 2013b; Hasam-Henderson et al., 2018; Rossetti et al., 2018). The observed reduced PV expression might result from altered PV-INs neurodevelopment (Cabungcal et al., 2013b; Barron et al., 2017), such as neuronal immaturity, which appears to be directly correlated to reduced PV expression (Gandal et al., 2012). This is of particular relevance from a functional perspective considering that reduced PV expression is considered as a surrogate biomarker for the activity of PV-INs. Oxidative stress appears also to be potentially associated with a reduction of PV-INs total number, as recently suggested by Steullet et al. (2018). These same mechanisms of excessive oxidative stress damaging mitochondria and PV-INs have been implicated in epilepsy, with evidence suggesting a causal link between the loss or dysfunction of PV-INs mediated by oxidative stress, and epileptic activity (Liang et al., 2000; Drexel et al., 2017; Kim and Kang, 2017; Gu et al., 2018; Wang et al., 2018). In fact, the regulation of ROS and oxidative stress has been proposed to have a potential therapeutic role for epilepsy (Eastman et al., 2020). For example, the antiepileptic treatment with cannabidiol (CBD) significantly reduced PV-INs downregulation, possibly preventing Ca^2+^ overloading during hyperactivity (Khan et al., 2018) and hence protecting neurons against mitochondrial oxidative stress (Hajnòczky et al., 2006).

The expected effects of PV-INs impairments or reduced PV expression are a decrease of PV-INs inhibitory postsynaptic currents (IPSC) onto excitatory neurons, which causes a reduced inhibitory control, and in turn excitation/inhibition imbalance (Caballero et al., 2020). This is supported by the notion that PV-INs-selective optogenetic suppression disinhibits cortical pyramidal neurons (Courtin et al., 2014) and increases excitatory neurons activity (Veit et al., 2017). In line with this, in juvenile mice models, the selective removal of one allele of the gene for GAD67 in PV-INs leads to a reduction in the synaptic transmission from PV-INs to pyramidal neurons and a consequent disinhibited pyramidal cell network (Lazarus et al., 2015).

In agreement with the above evidence, Inan et al. demonstrated that oxidative phosphorylation deficits lead to the inability of PV-INs to sustain their typical high-frequency firing, resulting in a disinhibited network with an imbalance of excitatory/inhibitory control (Inan et al., 2016) and network hyperexcitability.

Further confirmatory evidence includes the notion that administration of a mitochondrial fission activator, which induced mitochondrial fragmentation in PV-INs and resulting cell loss, triggers prolonged seizures (Kim and Kang, 2018). This is also consistent with numerous studies reporting the detrimental effect of experimental mitochondrial inhibition, or experimental metabolic stress, on the generation of fast-oscillatory gamma waves (summarized in Table 1).

**TABLE 1:** Results from systematic search on literature (PubMed) of the following keywords: Mitochondria AND Gamma oscillations. Only data that directly investigated the effect that mitochondrial/metabolic impairments exert on gamma oscillations have been included, criterion assessed based on information illustrated in titles/abstracts.

Authors and year	Type of study species	Method	Brain areas analyzed	Main conclusion
Hollnagel et al. (2020)	Rat brain slices	Increased lactate levels	Hippocampus	↓ Gamma oscillations
Bas-Orth et al. (2020)	Mouse brain slices	MCU knockout and knockdown	Hippocampus	↓ Gamma oscillations
Elzoheiry et al. (2020)	Rat brain slices	MCI inhibition (by rotenone)	Hippocampus	↓ Gamma oscillations
Berndt et al. (2018)	Rat brain slices	Induced mitochondrial dysfunction (by propofol)	Hippocampus	↓ Gamma oscillations
Robson et al. (2018)	Mouse brain slices	MC-IV and MCI inhibition (KCN and rotenone)	Hippocampus	↓ Gamma oscillations
Inan et al. (2016)	Mouse brain slices	Cox10 ablation	mPFC and Hippocampus	↑ Gamma oscillations
Galow et al. (2014)	Rat brain slices	Low glucose levels	Hippocampus	↓ Gamma oscillations
Lu et al. (2012)	Mouse brain slices	Low mitochondrial protonophores	Hippocampus	↓ Gamma oscillations
Kann et al. (2011)	Mouse/rat brain slices	MCI inhibition (by rotenone)	Hippocampus	↓ Gamma oscillations
Huchzermeyer et al. (2008)	Rat brain slices	Hypoxic conditions	Hippocampus	↓ Gamma oscillations

It is also worth considering the supportive role that the perineuronal net (PNN) exerts on PV-INs, which impacts their maturation and synaptic stability and also includes protection against oxidative stress (Cabungcal et al., 2013a). PNN impairment has been found to be implicated in neuropsychiatric conditions such as schizophrenia (Enwright et al., 2016) and Fragile X syndrome (Wen et al., 2018), with preliminary evidence of PNN alterations in BPAD (Steullet et al., 2018).

PV loss, in turn, affects mitochondrial structure (see studies listed in Table 2) suggesting that the relationship between mitochondrial and PV dysfunction is bidirectional. One possible factor contributing to this bidirectional relationship is related to the protective role of parvalbumin against mitochondrial Ca^2+^ overload, as proposed by Ruden et al., 2021. Several studies, using both *in vitro* and animal models, have assessed the effect on mitochondria of experimental manipulations of PV, demonstrating the close relation between PV-INs functioning and mitochondria, as summarized in Table 2. More in detail, it appears that PV knockout (PV^−/−^) in mice directly affects mitochondria, leading to morphological and density alterations in mitochondria of PV-INs (Janickova et al., 2020) and increased oxidative stress in PV-INs (Janickova and Schwaller, 2020). These findings overall support the idea that adequate PV presence is important to maintain a normal mitochondria structure and function.

**TABLE 2:** Results from systematic search on literature (PubMed) of the following keywords: Parvalbumin AND mitochondria. Only data that directly investigated the effect that parvalbumin impairments exert on mitochondria have been included, criterion assessed based on information illustrated in titles/abstracts.

Authors and year	Type of study/species	Method	Brain areas analyzed	Main conclusion
Janickova et al. (2020)	Mouse brain slices	PV knockout	SSC, mPFC, CA1, CA3, DG, cerebellum, and striatum	↑ Mitochondria volume and density
Janickova and Schwaller (2020)	Mouse brain slices	PV knockout	TRN; striatum	↑ Mitochondria volume and density
Lichvarova et al. (2019)	Rat cell cultures (CG4 OPCs)	PV-INs upregulation	N/a	↓ Mitochondrial volume
Lichvarova et al. (2018)	Madin-Darby canine kidney (MDCK) epithelial cells	PV-INs downregulation	N/a	↑ Mitochondria length and volume
Mendes et al. (2016)	Thyroid carcinoma cell lines	Forced PV expression	N/a	↓ Ca2+ inflow in the mitochondria
Henzi and Schwaller, (2015)	PV-negative MDCK cells	Ectopic expression of PV	N/a	↓ COX1 and mitochondrial volume
Chen et al. (2006)	Mouse brain slices	PV knockout	Cerebellum	↑ Mitochondrial volume and density
Meaetzler et al. (2004)	Mouse brain slices	PV upregulation	Striatum	↓ Mitochondrial volume
Chen et al. (2001)	Extensor digitorum longus of mice	PV knockout	N/a	↑ Mitochondria volume

Based on these pieces of evidence, it appears that mitochondrial impairment might not only lead to PV-INs impairments or downregulation but might be directly exacerbated by PV-INs disfunction. The effects that PV inhibition/deficit exerts on mitochondria might underlie the clinical deterioration characteristic of disease progression in some forms of BPAD. This idea is supported by the positive correlation between oxidative stress levels and disease severity (Sowa-Kućma et al., 2018), the association of higher levels of oxidative stress to worsening quality of life in patients (Nunes et al., 2018), and the efficacy of mitochondrial-targeted treatments in improving BPAD depressive symptoms (Mehrpooya et al., 2018). It is therefore plausible that mitochondrial abnormalities associated with mood disorders lead to PV-INs dysfunction and that this in turn disrupts neuronal network oscillation but also further contribute to mitochondrial damage, both exacerbating disease progression and overall worsening of disease severity.

### Alternating States of Neuronal Excitability in BPAD-Derived iPSCs Parallel Bipolar Behavioral Phenotypes

The above-described deficits in PV-INs and the resulting effects on neuronal excitability might play a role in the pathophysiology of mood instability characteristic of BPAD. Intrinsic alterations of neuronal excitability in BPAD have been described by Mertens et al. (2015) in hippocampal granule cells derived from BPAD patients using induced pluripotent stem cells (iPSCs) from reprogrammed fibroblasts. They reported a general hyperexcitable phenotype characterized by stronger Na^+^ channels activation, lower AP threshold, higher values of evoked and spontaneous AP frequencies, and maximal AP amplitude in BPAD-derived dentate gyrus granule cells. These data have been later confirmed by further evidence using iPSCs derived from lymphocytes of BPAD patients, showing that dentate gyrus-like granule cells were more hyperexcitable, with higher spontaneous and evoked AP firing rate compared to those of control subjects (Stern et al., 2018). Although GABAergic INs deficit was not directly demonstrated in this and related studies, we speculate that a hyperexcitable pattern might be exacerbated by a defective modulation by PV-INs on such neurons: anatomically, PV-INs axons in the dentate gyrus mainly innervate and form synapses on cell bodies and initial axonal segments of granule cells, and in normal conditions, they provide strong feedback and feedforward inhibition to these cells (Ribak et al., 1990; Sik et al., 1997; Houser, 2007; Pelkey et al., 2017), maintaining a proper inhibitory surround and directly regulating excitatory cell AP initiation (Hu et al., 2014) through their hyperpolarizing effect. It is plausible that functional or numerical deficit of PV-INs could directly affect granule cells excitatory potential, suggesting a possible exacerbating role of defective inhibitory interneurons in the hyperexcitable phenotype detected in BPAD by Mertens et al. (2015) and by Stern et al. (2018) although this hypothesis requires empirical testing. Extrapolating from the above-reported findings evidence and considering the higher presence of PV-INs in hippocampal CA1 and CA3 regions (Jinno and Kosaka, 2002; Bezaire et al., 2013) relative to the dentate gyrus (Ribak et al., 1990), we would expect even more marked alterations of neuronal excitability in these regions that also have an important affective modulation role. In more recent studies, CA3 pyramidal neurons derived from BPAD patients (using iPSCs) exhibited a greater frequency rate of induced and spontaneous activity and higher spike amplitude compared to controls-derived pyramidal neurons, which were associated with greater sodium and potassium currents (Stern et al., 2020a; Stern et al., 2020). Stern et al. (2020) also provided correlational evidence pointing to the overexpression of Voltage-Gated K^+^ channels (VGKCs) as a factor leading to the observed hyperexcitability; however, the evidence supporting the role of VGKCs as an underlying mechanism appears inconclusive. The authors suggested that VGKC functional changes might be an indirect, or even homeostatic compensatory, mechanism resulting from neuronal intrinsic hyperactivity. Further research is warranted to test the possibility that PV-INs dysfunction might contribute to the observed hyperexcitable patterns.

Lithium reversed the hyperexcitability of hippocampal neurons derived from lithium-responsive BPAD patients (Mertes et al., 2015). This hyperexcitable phenotype of iPSCs neurons, also characterized by exquisite sensitivity to lithium modulation, might have a phenomenological correspondence in the elevated mood, euphoria, and hyperactivity that characterizes manic episodes in BPAD, for which lithium displays excellent therapeutic and prophylactic properties (Cade, 1949; Hartigan, 1963; Kessing et al., 2018).

However, while the hyperexcitable lithium-responsive BPAD-derived iPSCs represent an attractive biological model for the manic dimension of BPAD, it is important to consider that mania is only one of the multiple aspects constituting the complex clinical phenotype of BPAD and related disorders in the Bipolar spectrum. Patients suffering from BPADs spend more of their symptomatic time in a state of depression relative to time spent with mania. Subsyndromal, rapid cycling, and mixed affective symptoms predominate over the full, syndromal-level, major affective episodes. Mood instability and subthreshold depression are central and key elements of the BPAD behavioral phenotype, and an optimal cellular model for BPAD would need to present characteristics that reflect as closely as possible the cyclic, mixed, and highly unstable nature of the Bipolar affective phenotype.

Recent studies following from the seminal work by Mertens et al. (2015) by the same group of authors provided intriguing and important signals in that direction.

First, hyperexcitability was noticed only in neurons derived from lithium-responsive patients, while CA3 and DG neurons derived from nonlithium-responsive BPAD were not hyperexcitable (Stern et al., 2018; 2020). This indicates that BPAD-derived neurons do not present homogenous characteristics in terms of both intrinsic excitability and pharmacological responsiveness. This is paralleled by clinical data that indicate a preferential therapeutic effect of lithium on mania, while its efficacy on depression, mixed, rapid cycling, and subsyndromal states is lower.

Second, within the same iPSCs culture, BPAD-derived cells were not purely characterized by a hyperexcitable pattern (Stern et al., 2020): among neurons derived from nonlithium-responsive BPAD patients, many displayed a hypoexcitable phenotype, and others appeared normally excitable. Although these were data based on computationally simulated BPAD-derived neurons, we feel that the finding of coprevalence in the same BPAD cell culture of both hypo- and hyperexcitable cells, with greater diversity in neuronal excitability compared to controls’ neurons, is worth noting and potentially translatable.

These observations might indicate a potential vulnerability of hippocampal interneurons of BPAD patients in terms of intrinsic instability of neuronal excitability. The existence of multiexcitatory states observed in iPSCs cultures derived from patients’ neurons opens to the speculative idea that a similar heterogeneity of neuronal excitatory states might also exist *in vivo* in the BPAD patients’ brain. This possibility would have a logical correspondence in the clinical mood instability experienced by patients alternating through episodes of hyper- (mania) or hypo- (depression) activity, which could also manifest simultaneously during mixed states (Fagiolini et al., 2015; Solé et al., 2017).

We further propose that the dysfunction or downregulation of PV-INs, consequential to neurometabolic and mitochondrial deficits, might represent a mechanism leading to loss of excitatory network stabilization, which might contribute to, or aggravate, the intrinsically unstable excitatory pattern characteristic of the BPAD brain. The combined effect of the intrinsic cooccurrence of hyper- and hyponeuronal excitability states and of a defective regulatory system secondary to downregulated GABAergic inhibitory control might cumulatively contribute to the instability and cycling of affective states with opposite polarities.

Extrapolating from previously reported observations of a bidirectional relationship between altered neuronal excitability, directly induced by PV deficits, and mitochondrial damage, it is plausible to expect that states of hyperexcitability underlying mania are also leading to mitochondrial stress and impairment. This resonates well with converging data suggesting that accumulation of manic episodes (but not depressive episodes) through the lifetime of BPAD patients is associated with neuroprogression which manifests with progressive cognitive and functional decline and overall worsening of disease severity (López-Jaramillo et al., 2010; Passos et al., 2016).

### Novel Pharmacological Approaches

Based on the proposed model, the recent development of pharmacological treatments that selectively target PV-INs is highly promising. Enhancement of PV-INs-dependent neurotransmission could potentially contribute to stabilizing the abnormal excitatory pattern by virtue of reduced inhibitory control, ultimately resulting in improvement of mood instability.

K_v_3, a specific subunit of K^+^ channels, is specifically expressed in PV-INs, in particular K_v_3.1 (Weiser et al., 1995; Sekirnjak et al., 1997) and K_v_3.2 (Chow et al., 1999). They are implicated in the generation of fast-spiking firings by PV-INs (Erisir et al., 1999; Lien and Jonas, 2003) and they modulate the synchronization of cortical circuits, neuronal excitability (Rudy et al., 1999), and the generation of the brain’s oscillatory rhythms (Joho et al., 1999; Espinosa et al., 2008). These data explain the therapeutic potential of targeting K_v_3 subtypes in order to modulate PV-INs activity, the role that has already been significantly demonstrated in preclinical studies, with the use of K_v_3.1- and K_v_3.2-positive modulators, showing increased PV-INs firing frequencies (Boddum et al., 2017) and rescuing their fast firing phenotype after its impairment in conditions of K_v_3 blockade (Rosato-Siri et al., 2015). This modulation might be therefore a promising therapeutic tool in disorders associated with dysfunctions of inhibitory controls and unstable neuronal excitability, such as affective disorders. Recent pieces of evidence highlighted the potential role of this modulation in MDD and in BPAD, with preclinical studies that demonstrated that reduced K_v_3 in PV-INs in the dentate gyrus induced depression phenotypes (Medrihan et al., 2020) and that K_v_3.1- and K_v_3.2-positive modulators are able to reverse and prevent manic behaviors in mouse models (Parekh et al., 2018). For these reasons, K_v_3.1- and K_v_3.2-positive modulators are now being assessed in clinical trials and appear to be safe for human use (National Library of Medicine (U.S), 2019). These compounds have been tested in a human experimental model of schizophrenia, with promising results (Deakin et al., 2019). Ongoing clinical trials are now assessing directly the efficacy in schizophrenic patients (National Library of Medicine (U.S), 2017). The availability of compounds targeting selectively PV-INs would make them attractive as potential PET radioligands candidates that could enable direct visualization of these neuronal populations *in vivo*. Currently, available PET ligands for imaging the GABA system are ^11^C-Flumazenil and ^11^C-Ro15-4513, in which the first unselectively targets the GABA-A benzodiazepine receptor subtypes and the second is highly selective for the five subtypes (Lingford-Hughes et al., 2002; Maeda et al., 2003). Unfortunately, these GABA receptor subtypes are not exclusively expressed on subpopulations of GABAergic interneurons and this prevents the use of these tracers for separating PV-INs from other GABAergic interneurons. Furthermore, focusing on GABA receptors would not directly inform on PV-INs density. Integration of direct assessment of GABAergic neurons *in vivo*, with other procedures such as MEG, capable of measuring neuronal oscillations (e.g., gamma oscillations) in deeper brain structures, would enable us to study the effects of PV-INs loss and subsequent functional alterations. To our knowledge, no K_v_3 PET ligands have yet been developed, although ongoing work on a PET tracer for K_v_1 ligands has been reported (Brugarolas et al., 2018).

## Conclusion

In conclusion, the neurobiological evidence reported suggests a causal relationship between mitochondrial deficits and PV-INs dysfunction/downregulation in BPAD, which would consequentially contribute to or aggravate intrinsic neuronal excitability alterations, leading to cycling between mood states and to a mood instability phenotype, which is characteristic of more severe, and less treatment-responsive forms of BPAD. We reported meta-analytical evidence of postmortem downregulation of PV-INs in BPAD and described the direct detrimental effects of mitochondrial dysfunction on gamma oscillations and of PV-INs loss on mitochondria. These observations, taken together, suggest the existence of a bidirectional relationship between mitochondrial dysfunction and damage to the PV-INs system, which result in a vicious circle of progressive exacerbation of mitochondrial defects and functional neuronal alterations, leading to neuroprogression, and accumulation of mood instability. These pieces of evidence suggest the therapeutic potential of targeting PV-INs using novel promising compounds that could form the basis of both therapeutic drugs and novel selective PET ligands. Although our work has focused on BPAD to validate the mechanistic links we proposed, targeting the PV-INs system would have therapeutic potential for the broader spectrum of conditions associated with mood instability, and more widely for the numerous other neurological and psychiatric conditions characterized by neurometabolic and neuroexcitability, such as epilepsy, neurodegeneration, anxiety disorders, MDD, and schizophrenia.

## Data Availability

The original contributions presented in the study are included in the article/Supplementary Material; further inquiries can be directed to the corresponding author.
