# Supplementary material for: The Neurometabolic Basis of Mood Instability: The Parvalbumin Interneuron Link—A Systematic Review and Meta-Analysis
Source: Front Pharmacol. 2021 Sep 20;12:689473. doi: 10.3389/fphar.2021.689473 (PMC8488267; doi:10.3389/fphar.2021.689473)
Supplement: Supplementary file 1 [file DataSheet1.PDF]

Supplementary Tables

Authors and year	Sample size BPAD/HC	BPAD type	Brain region analyzed	Outcome measure	Significant findings (BPAD relative to HCs)	Marker used	Fixation method used
Steullet et al., 2018	15/20	Unspecified	TRN	PV-INs total number	↓in TRN in BPAD ($p<0.0001$)	Clone PARV-19	4% PFA
Pantazopoulos et al., 2010	11/15	Unspecified	Amygdala LN, BN, AB, CO	PV-INs total number	↔ in LN ($p=0.51$)<br>↔ in BN ($p=0.94$)<br>↔ in AB ($p=0.99$)<br>↔ in CO ($p=0.28$)	Clone PARV-19	4% PFA
Konradi et al., 2011	14/18	Mostly BPAD 1 (13/14)	Hippocampus (CA1,2/3,4)	PV-INs total number	↓ in BPAD ($p<=0.029$)	Clone PARV-19	4% PFA
Pantazopoulos et al., 2007	10/16	Unspecified	EC-L, EC-I, EC-C, EC-R	PV-INs total number	↓ in all EC in BPAD ($p=0.02$)	Clone PARV-19	4% PFA

Supplementary table 1: Summary of studies assessing total number on PV-INs. Arrows represent: ↓=significant reduction; ↔= no significant changes in the BPAD group compared to controls; ↑=significant increase. The last column in the right side lists the different fixation methods used by the included studies, referring to either 4% paraformaldehyde (PFA) or paraffin wax embedded.

**Authors and year**	**Sample size BPAD/HCs**	**BPAD type**	**Brain region analyzed**	**Outcome measure**	**Significant findings ($p<0.05$) (BPAD relative to HCs)**	**Marker or technique used**	**Fixation method used**
Pantazopoulos et al., 2010	11/15	Unspecified	Amygdala LN, BN, AB, CO	Cell density (cells/mm^3)	↓ in LN in BPAD (p=0,03)<br><br>↔ in BN (p=0.94)<br><br>↔ in AB (p=0.99)<br><br>↔ in CO (p=0.28)	Clone PARV-19	4% PFA
Wang et al., 2011	13/17	BPAD-1	All EC	Num density (cells/mm^2)	↓ in all ECx in BPAD ($p \leq 0.05$)	Clone PARV-19	4% PFA
Pantazopoulos et al., 2007	10/16	Unspecified	EC-L, EC-I, EC-C, EC-R	Num density (cells/mm^3)	↓ in all EC in BPAD ($p< 0.05$)	Clone PARV-19	4% PFA
Beasley et al., 2002	15/15	Unspecified	DLPFC	Cell density (cells/mm^2)	↔ in DLPFC ($p>0.05$)	clone PA-235	Paraffin
Reynolds et al., 2002	15/15	Unspecified	DLPFC and EC	Tot cortical density (cells.mm^{-2})	↔ in DLPFC ($p>0.05$)<br><br>↔ in EC ($p>0.05$)	clone PA-235	Paraffin

Zhang et al., 2002	14/15	Unspecified	Hippocampus (DG, CA1-CA4)	Relative density (cells/mm^2)	↓ in CA1 in BPAD ($p<0.05$)<br>↔ in other regions	clone PA-235	Paraffin
Cotter et al., 2002	15/15	Unspecified	ACC	Laminar density (cells/mm^2)	↔ in ACC ($p>0.05$)	clone PA-235	Paraffin
Alcaide et al., 2019	13/11	Unspecified	DLPFC	Num density (cells/mm^2)	↔ in DLPFC ($p>0.05$)	polyclonal guinea pig anti-PV antibody	4% PFA
Steullet et al., 2018	15/20	Unspecified	TRN	Num density (cells/mm^3)	↓ in TRN in BPAD ($p<0.0001$)	clone PARV-19	4% PFA
Sakai et al., 2008	5/5	Unspecified	BA9 (PFC)	Cell density (cells/mm^2)	↔ in BA9 ($p>0.05$)	polyclonal anti-PV	Paraffin

Supplementary table 2: Summary of studies assessing density of PV-INs. As displayed, in some studies, cell density has been investigated using different unit of lengths, such as cells/mm^2 (displayed also as cells.mm^{-2}) and cells/mm^3. ↓=significant reduction; ↔= no significant changes in the BPAD group compared to controls; ↑=significant increase. In addition to the studies included in the quantitative analysis, here are included also the studies available for the qualitative but not for the quantitative analysis (n=1; Cotter *et al.*, 2002). The last column in the right side lists the different fixation methods used by the included studies, referring to either 4% paraformaldehyde (PFA) or paraffin wax embedded.

**Authors and year**	**Sample size BPAD/HC**	**BPAD type**	**Brain region analyzed**	**Outcome measure**	**Significant findings (p<0.05) (BPAD relative to HCs)**	**Technique used**
Volk et al., 2016	25/25	Unspecified	BA9 (PFC)	PV mRNA ratio (2^{-dCT})	↓ in BA9 in BPAD (p=0.001)	qPCR analysis
Sibille et al., 2011	12/12	BPAD-1	DLPFC	PV mRNA rel express (2^{-dCT})	↓ in DLPFC in BPAD (p=0.004)	qPCR analysis
Konradi et al., 2011	14/14	Mostly BPAD 1 (13/14)	Hippocampus (CA1,2/3,4)	PV mRNA expression (2^{-dCT})	↔ in CA1 (p>0.05)<br>↓ in CA2/3 in BPAD (p<=0.026)<br>↓ in CA4 in BPAD (p<=0.007)	qPCR analysis
Fung et al., 2014	31/34	Unspecified	DLPFC and OFC	PV mRNA expression (2^{-dCT})	↔ in DLPFC (p>0.05)<br>↔ in OFC (p>0.05)	qPCR analysis
Chung et al., 2018	40/40	Unspecified	DLPFC	PV mRNA expression (2^{-dCT})	↓ in DLPFC in BPAD (p=0.002)	qPCR analysis

Supplementary table 3: Summary of studies assessing PV mRNA levels. Arrows represent: ↓=significant reduction; ↔= no significant changes in the BPAD group compared to controls; ↑=significant increase. In addition to the studies included in the quantitative analysis, here are included also the studies available for the qualitative but not for the quantitative analysis (n=1; Fung *et al.*, 2014).
